# Epidemiology of Pufferfish Poisoning in Japan: Trends and Insights from 2004 to 2023

**DOI:** 10.31662/jmaj.2025-0543

**Published:** 2026-07-10

**Authors:** Yoshiro Hadano, Yuichiro Tanaka, Satoshi Ohno

**Affiliations:** 1 Division of Infection Control and Prevention, Shimane University Hospital, Izumo, Shimane, Japan; 2 Faculty of Medicine, Shimane University, Izumo, Shimane, Japan; 3 Clinical Research Center, Shimane University Hospital, Izumo, Shimane, Japan

**Keywords:** food poisoning, tetrodotoxin, pufferfish, Japan

## Abstract

**Introduction:**

Pufferfish poisoning caused by tetrodotoxin remains a serious and potentially fatal public health concern in Japan, despite stringent regulations governing its traditional consumption.

**Methods:**

Epidemiological trends in pufferfish poisoning over the past two decades were retrospectively analyzed using data from the Ministry of Health’s “Foodborne Illness Statistical Data” reports between January 2004 and December 2023. Trends in poisoning incidents, affected patients, and fatalities were assessed as primary outcomes. The regional distribution, seasonal patterns, and contributory risk factors, particularly domestic preparation, were also investigated.

**Results:**

Between 2004 and 2023, Japan documented 450 incidents of pufferfish poisoning, involving 622 individuals and resulting in 17 deaths. The annual incidence rates for pufferfish poisoning events and affected patients declined by 5% and 8%, respectively. Poisonings occurred more frequently during winter, though summer incidents—including fatalities—were also documented. The majority of incidents, affected individuals, and deaths were linked to domestic preparation. Western Japan reported the highest number of events and incidence rates per 1,000,000 population.

**Conclusions:**

Although pufferfish poisoning events declined over the past two decades, fatal outcomes persisted, primarily because of improper handling during domestic preparation. Enhancing awareness of the risks associated with home preparation remains essential to mitigate tetrodotoxin-related poisonings.

## Introduction

Tetrodotoxin represents the most prevalent marine biotoxin associated with foodborne poisoning in Japan, posing a substantial public health concern ^(1)^. Pufferfish, known as “fugu” in Japanese, possess tetrodotoxin, which is produced by marine bacteria and accumulates in pufferfish organs through the food chain^2^. Even minute quantities of this toxin can induce severe or fatal poisoning ^(2), (3)^. Tetrodotoxin primarily blocks voltage-gated sodium channels, thereby impairing nerve and skeletal muscle function while largely sparing cardiac activity ^(4), (5)^. In pufferfish, particularly high toxin concentrations are present in the liver and ovaries, although some species also harbor tetrodotoxin in the muscle or skin ^(6)^. Tetrodotoxin is heat-resistant and is not destroyed through cooking or washing. Clinical symptoms typically manifest within 6 hours of ingestion and involve sensory disturbances such as perioral paresthesia—numbness around the lips, tongue, and distal extremities ^(7)^. Additional symptoms may develop within hours, progressing to paralysis and respiratory failure if not promptly identified and treated ^(4)^. Diagnosis is based on clinical presentation and can be confirmed after detecting tetrodotoxin in the blood, urine, or residual food. No specific antidote exists, and treatment is primarily supportive, including gastric lavage, fluid administration, and ventilatory support as required^4^. Symptoms generally peak within 6-8 hours, and survival beyond 24 hours is typically associated with recovery ^(1), (2), (3), (4)^.

Japan is among the few countries in which pufferfish is consumed and, despite its toxicity, remains one of the largest consumers of this delicacy; the fish has become an integral part of the nation’s culinary heritage ^(4), (8)^. Preparing pufferfish demands specialized knowledge and expertise, as improper handling can lead to severe poisoning. In Japan, fugu is meticulously prepared by licensed chefs to ensure safe consumption. Nonetheless, poisoning incidents and fatalities continue to occur, often associated with improper domestic preparation ^(9)^.

Tetrodotoxin poisoning constitutes a major health concern in Japan and other Asian countries. However, reports of tetrodotoxin-related incidents have also emerged globally, including in Australia, Bangladesh, Taiwan, the United States, and the Mediterranean region ^(10)^. Importantly, these incidents are not limited to pufferfish consumption, as tetrodotoxin has been detected in a variety of marine and terrestrial organisms, such as gastropods, crabs, and amphibians ^(11)^.

Previous reports, including Toda et al. ^(8)^, have described trends in foodborne poisoning cases in Japan from 1989 to 2010, including those caused by pufferfish. However, information on more recent trends remains limited. Moreover, some emerging issues may have been overlooked amid ongoing public health measures. Despite its clinical significance, epidemiological data on pufferfish poisoning remain insufficient. In this study, we aimed to clarify the epidemiological trends of pufferfish poisoning in Japan over the past two decades.

## Materials and Methods

This retrospective study was conducted from January 2004 to December 2023. In Japan, physicians are legally obligated to report suspected incidents of all food poisoning cases, including pufferfish poisoning, to local public health centers according to the Food Sanitation Act. Food poisoning is defined as “patients who have been poisoned, or are suspected of being poisoned, by food, additives, utensils or containers, and packaging” ^(12)^. Furthermore, public health centers subsequently conduct investigations to determine the source of contamination, including the origin and the implicated food or pathogen. When a report of foodborne illness is received from an individual or restaurant, the event is promptly investigated and classified as food poisoning ^(12)^. Diagnosis is primarily based on the patient’s history of pufferfish consumption and clinical observation of tetrodotoxin toxicity symptoms. Alternatively, confirmation may be obtained through the detection of tetrodotoxin in the patient’s urine, serum, or residual fish samples. Findings from the investigation are subsequently reported to the Ministry of Health, Labour and Welfare (MHLW).

Data on pufferfish poisoning events were obtained from the MHLW website, specifically from the Foodborne Illness Statistical Data reports spanning January 2004 to December 2023 ^(12), (13)^. These datasets included monthly totals of food poisoning incidents caused by food as well as by pathogenic agents. Although pufferfish toxin is classified as a naturally occurring animal toxin, the dataset did not provide specifications on toxin type. Available data also included the number of affected patients and fatalities per year, as well as information by sex, age group, causative food, and prefecture. Cases of pufferfish poisoning in this study were identified using the MHLW’s monthly aggregated tables, particularly those categorized as “pufferfish” under the causative food classification. The analysis focused on the annual incidence of tetrodotoxin poisoning, as well as the number of affected patients and fatalities in Japan. The annual incidence rates by prefecture and the national average per 1,000,000 population were calculated. In addition, the location of each event was classified as home-based or non–home-based. For each year, the numbers of incidents, affected individuals, and deaths attributable to home-based poisoning were extracted, and their proportions relative to all pufferfish poisoning cases were calculated. Additionally, data on domestic catch and import volumes of pufferfish (in tonnes), along with annual population statistics, were retrieved from the MHLW “e-Stat” portal ^(14)^. Total annual pufferfish consumption was defined as the sum of domestic catch and import volumes. According to the Ethical Guidelines for Epidemiological Research issued by the Japanese MHLW, ethical review was not required, as the study utilized anonymized and de-identified data. Consequently, ethical and institutional review board approval was not obtained.

### Statistical analyses

A negative binomial model was employed to assess the association between the annual trends in tetrodotoxin poisoning events and total pufferfish consumption. The logarithm of the population size was included as an offset variable to account for population differences over time. Annual data from 2004 to 2023 were used to aggregate the number of household incidents, affected individuals, and deaths. The percentage (rate) of each category relative to the total was calculated.

Poisson regression models were applied to evaluate the temporal trends in the annual number of home-based incidents and patients from 2004 to 2023. The year was treated as a continuous explanatory variable to assess changes over time. To account for differences in exposure, the logarithm of the total number of incidents or affected individuals was included as an offset term in each model. Two-tailed p-values < 0.05 were considered statistically significant. Relevant packages in R were used to visualize the distribution of pufferfish poisoning events across Japan. Geographical data for Japan’s prefectures were obtained using the “rnaturalearth” package ^(15)^. Statistical analyses were conducted using R, version 4.3.0 (The R Foundation for Statistical Computing, Vienna, Austria).

## Results

Between 2004 and 2023, a total of 450 pufferfish poisoning incidents were reported in Japan, involving 622 patients and 17 fatalities. Overall, the number of incidents declined across the two- decade period (Figure 1).

**Figure 1.**
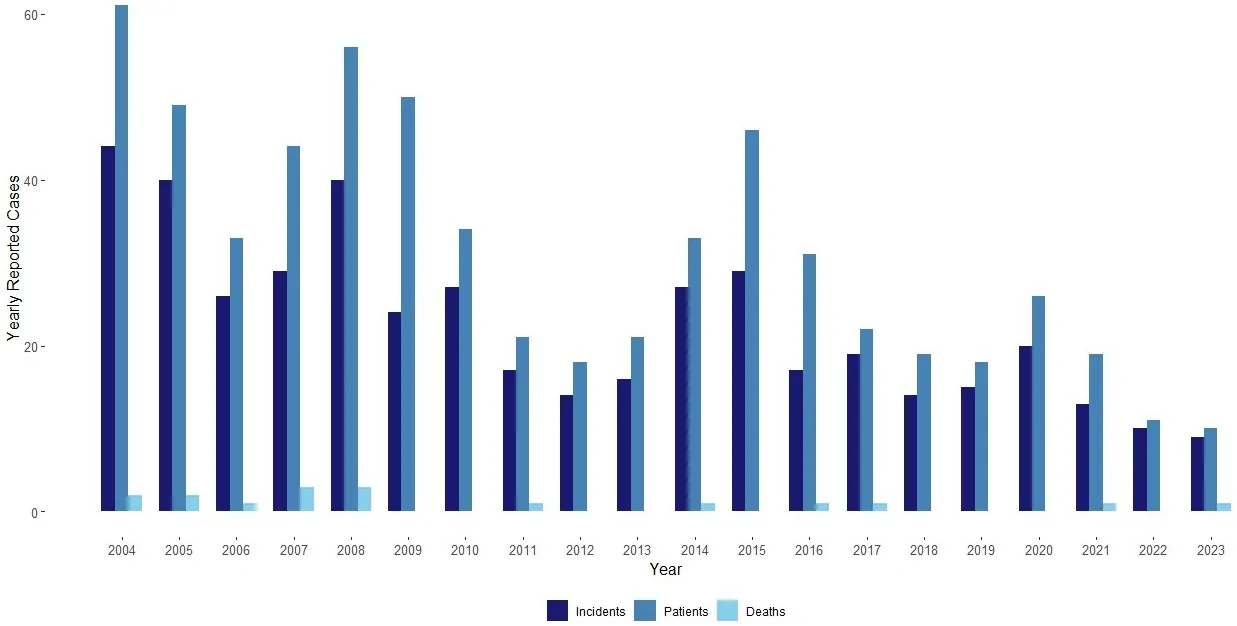
Total numbers of incidents, affected individuals, and fatalities due to pufferfish poisoning (2004-2023).

A marked male predominance was observed among reported events (72.2%). Most events (78.6%) occurred in individuals aged ≥50 years, with the highest numbers recorded in the 60-69 (n = 190), ≥70 (n = 171), and 50-59 (n = 139) year age groups (Table 1). During the same period, 17 fatalities were reported, with a pronounced predominance among males (94.1%). All fatalities occurred in individuals aged ≥40 years, with no fatalities documented among those under 40 years of age. The overall event fatality rate was 2.7%.

**Table 1.** Demographic Distribution of Reported Events and Associated Mortality (2004-2023).

Age group (years)	Reported events			Associated mortality		
	Total	Male	Female	Total	Male	Female
0-4	1	1	0	0	0	0
5-9	1	1	0	0	0	0
10-14	3	2	1	0	0	0
15-19	13	8	5	0	0	0
20-29	11	7	4	0	0	0
30-39	39	22	17	0	0	0
40-49	52	34	18	2	1	1
50-59	139	112	27	3	3	0
60-69	190	139	51	7	7	0
70+	171	121	50	5	5	0
Unknown	2	2	0	0	0	0
**Total**	622	449	173	17	16	1

The number of pufferfish poisoning incidents and affected individuals was higher during the winter and lower in the summer, although fatal events were also reported in the summer (Figure 2). Over the past two decades, domestic catch and import volumes of pufferfish declined, resulting in an approximate one-third reduction in total annual consumption (Supplementary Table 1).

**Figure 2.**
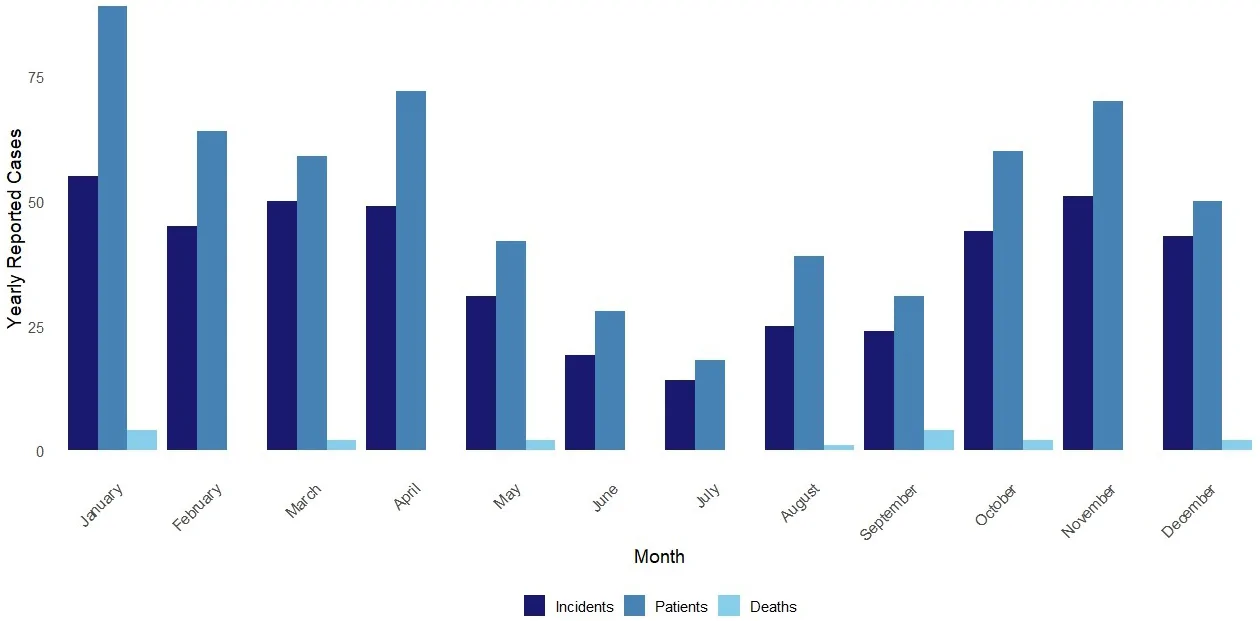
Seasonal distribution of diagnosed pufferfish poisoning events in Japan (2004-2023).

The number of incidents, affected individuals, and fatalities declined over the past two decades. A significant annual decline of 5% was observed in the incidence rate (adjusted incidence rate ratio [IRR]: 0.95; 95% confidence interval [CI]: 0.91-0.99; p < 0.01). The number of affected individuals showed a comparable annual reduction of 8% (adjusted IRR: 0.92; 95% CI: 0.86-0.97; p < 0.01). Although the decline in fatalities was not statistically significant, a downward trend was evident (adjusted IRR: 0.90; 95% CI: 0.73-1.11; p = 0.28) (Table 2).

**Table 2.** Parameter Estimates from the Negative Binomial Regression Model Assessing Patient Volume, Incident Rates, and Mortality.

Model	Variables	Coefficient	95% CI	p-Value
Incident count	Year	–0.05	0.95 (0.91-0.99)	<0.01
	Pufferfish consumption	0.00	1.00 (0.99-1.00)	0.69
				
Patient count	Year	–0.08	0.92 (0.86-0.97)	<0.01
	Pufferfish consumption	0.00	1.00 (0.99-1.00)	0.53
				
Death count	Year	–0.11	0.90 (0.73-1.11)	0.28
	Pufferfish consumption	0.00	1.00 (0.99-1.00)	0.93

CI: confidence interval.

The majority of the events, including affected individuals and fatalities, occurred in households, accounting for 76.9%, 69.5%, and 70.6%, respectively (Supplementary Table 2). Over the past two decades, household-associated incidence and affected individuals rates exhibited an upward trend (Supplementary Table 3), particularly when data were aggregated into 5-year intervals. Between 2004-2008 and 2019-2023, the proportion of household incidents increased from 73.6% to 85.1%, while the proportion of household patients rose from 67.5% to 85.7%.

Over the past two decades, pufferfish poisoning events were concentrated in western Japan. The five prefectures with the highest incidence rates were Hiroshima (n = 94), Yamaguchi (n = 50), Fukuoka (n = 46), Nagasaki (n = 46), and Hyogo (n = 41) (Supplementary Figure 1). Supplementary Figure 2 presents the average incidence rates per 1,000,000 population across Japanese prefectures during this period. The highest rates were observed in Tottori (1.88), Yamaguchi (1.72), Hiroshima (1.65), Nagasaki (1.60), and Shimane (1.14). Overall, incidents were more frequently reported in western Japan (Figure 3).

**Figure 3.**
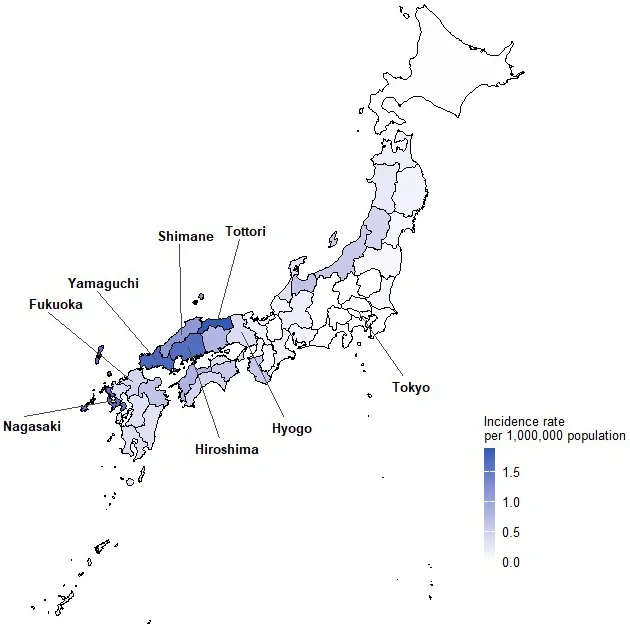
Geographic heat maps showing annual pufferfish poisoning events per 1,000,000 population across Japan (2004-2023).

## Discussion

This study outlines recent trends in pufferfish poisoning in Japan. Although the number of events has declined over the past two decades, incidents continue to occur. In recent years, approximately 20 incidents have been reported annually in Japan, with a few resulting in fatalities. Additionally, the proportion of poisonings linked to home preparations has increased during this period.

Japan has experienced numerous incidents of tetrodotoxin poisoning from pufferfish, with reports dating back to 1965 ^(1)^. That year, 106 incidents involving 152 patients and 88 deaths were recorded, reflecting an exceptionally high mortality rate of 57.9%. The decline in events over time suggests that public health measures and awareness campaigns implemented by national and local public health organizations have been effective. Over the past two decades, both domestic catch and import volumes of pufferfish have decreased, resulting in an approximate one-third reduction in total annual consumption. Concurrently, the proportion of farm-raised pufferfish—many of which are known to exhibit lower toxicity—has increased in recent years ^(16)^. These factors, including shifts in dietary habits and the broader adoption of cultivated pufferfish stocks, are considered to have contributed to the decline in pufferfish poisoning. However, as noted below, many incidents still occur in domestic settings, involving fish caught and prepared directly by the affected individuals themselves, family members, relatives, or friends.

In Japan, tetrodotoxin poisoning has occurred most frequently during winter, the peak season for pufferfish consumption, and has been most frequently reported in western Japan, particularly along the coastal areas of the Seto Inland Sea. Previous reports from 1989 to 2010 identified Hiroshima, Hyogo, Yamaguchi, and Fukuoka prefectures as areas with frequent occurrences ^(5)^. In the present study, the seasonal trend of elevated winter incidence persisted; however, high incidence rates per million population were also observed in the San’in region—including Tottori and Shimane prefectures —as well as in Nagasaki prefecture. Our findings based on population-adjusted rates suggest that the San’in region is a high-incidence area—potentially more so than previously recognized.

A notable observation in the present study was the annual rise in tetrodotoxin poisonings associated with home-prepared pufferfish. Each prefecture operates a licensing system for pufferfish chefs, which has played a key role in reducing the frequency of intoxications. Although the number of poisonings has significantly declined over the past two decades, most incidents and fatalities have stemmed from improper handling by unlicensed individuals in domestic settings—a proportion that has steadily increased. Licensed chefs possess specialized expertise in the safe preparation of fugu. Therefore, public education on the risks of preparing pufferfish at home remains essential to further mitigate these preventable outcomes.

From a global epidemiological perspective, tetrodotoxin poisoning caused by pufferfish is not only confined to Asian countries such as Japan but represents an international concern. Globally, >3,000 instances have been documented in the literature ^(17)^. The geographic spread of this toxin is believed to be linked to rising global water temperatures ^(18)^. Although few long-term epidemiological studies exist, numerous outbreaks have been reported across multiple countries. Similar to Japan, Bangladesh experienced recurrent poisonings between 1998 and 2008, including 141 reported events in 2008 ^(19), (20)^. Taiwan recorded several occurrences, including six intoxication events and one death in 2001 ^(21)^. Outside Asia, Australia reported 11 poisonings during 2001-2002 ^(22)^, while the Mediterranean region (Egypt and Israel) reported 13 events between 2005 and 2008 ^(23)^. In the US, tetrodotoxin poisoning linked to pufferfish imported from Japan was reported in California in 1996 ^(24)^, followed by additional incidents in Chicago in 2007 ^(25)^. These reports reflect the expanding geographic distribution of pufferfish-related intoxications, underscoring the growing global concern regarding tetrodotoxin exposure. They reinforce the need for heightened awareness, stricter regulations, and enhanced preventive measures to safeguard public health.

This study has several limitations. First, not all incidents were reported under the Food Sanitation Act, representing a concerning gap in surveillance. Although data from food poisoning outbreaks investigated by public health centers were included, the extent of event capture may have varied depending on the response and reporting practices of individual centers. Furthermore, some cases reported in conference abstracts or medical journals may be missing from the public health data owing to misclassification or a failure to report them to local authorities. Consequently, the true number of intoxications may have been underestimated. Second, the findings may not be generalizable to other countries or regions with differing epidemiological profiles or healthcare systems. Third, data on fish consumption by prefecture, the number of licensed fugu chefs, and their annual trends were unavailable due to limited dataset access. Finally, in this study, we did not examine clinical details, such as symptom progression, the species of pufferfish, or specific tissues consumed by affected individuals, as this information was unavailable in the databases used. In addition, because the datasets lacked information on the specific toxins involved, we could not distinguish between tetrodotoxin and saxitoxins in individual cases. While reports of pufferfish poisoning outbreaks exist, few recent studies have comprehensively analyzed the long-term epidemiology of tetrodotoxin poisoning in Japan. Despite the persistence of incidents involving home-prepared pufferfish, such longitudinal investigations remain rare. Consequently, our findings provide novel and valuable insights into this public health concern.

## Conclusions

This epidemiological study of pufferfish poisoning in Japan over the past two decades demonstrates that, although the overall incidence has significantly declined, fatal events continue to occur sporadically—primarily in instances involving home preparation. When adjusted for population, our findings suggest that the San’in region may also be a high-incidence area—possibly higher than that previously thought. Further research is warranted to achieve a more comprehensive understanding of the epidemiological characteristics of this health concern. These findings have important implications for public health policy and implementation in Japan. Targeted initiatives should prioritize raising awareness of the risks associated with domestic preparation of pufferfish.

## Article Information

### Author Contributions

Conceived the study, collected and interpreted the data, drafted the manuscript, and revised the manuscript for important intellectual content: Yoshiro Hadano. Conceived the study and collected and interpreted the data: Yuichiro Tanaka. Conceived the study and revised the manuscript for important intellectual content: Satoshi Ohno. All authors contributed to data acquisition and reviewed, discussed, and approved the final manuscript.

### Conflicts of Interest

None

### IRB Approval Code and Name of the Institution

This study utilized anonymized and de-identified data; therefore, ethical review was not required in accordance with the Ethical Guidelines for Epidemiological Research issued by the Japanese Ministry of Health, Labour and Welfare. Thus, ethical and institutional review board approval was not obtained for this investigation.

### Informed Consent

This study utilized anonymized and de-identified data; therefore, the requirement for informed consent was waived.

## Supplement

Supplementary Material
