# Supplementary material for: Epidemiology of Pufferfish Poisoning in Japan: Trends and Insights from 2004 to 2023
Source: JMA J. 2026 Jul 10;9(5):1224–30. doi: 10.31662/jmaj.2025-0543 (PMC13639586; doi:10.31662/jmaj.2025-0543)

Supplementary Table 1. Total Number of Incidents, Patient Cases, and Deaths of Home-based Poisoning Cases from 2004 to 2023.

Year	Number of Incidents	Number of Patients	Number of Deaths	Number of Home-based Incidents	Number of Home-based Patients	Number of Home-based Deaths	Home-based Incidents Ratio (%)	Home-based Patients Ratio (%)	Home-based Deaths Ratio (%)
2004	44	61	2	32	40	1	72.7	65.6	50.0
2005	40	49	2	33	40	2	82.5	81.6	100.0
2006	26	33	1	18	22	1	69.2	66.7	100.0
2007	29	44	3	20	24	3	69.0	54.5	100.0
2008	40	56	3	28	38	2	70.0	67.9	66.7
2009	24	50	0	16	20	0	66.7	40.0	0.0
2010	27	34	0	21	26	0	77.8	76.5	0.0
2011	17	21	1	13	15	0	76.5	71.4	0.0
2012	14	18	0	12	15	0	85.7	83.3	0.0
2013	16	21	0	12	15	0	75.0	71.4	0.0
2014	27	33	1	20	24	1	74.1	72.7	100.0
2015	29	46	1	21	25	0	72.4	54.3	0.0
2016	17	31	0	13	18	0	76.5	58.1	0.0
2017	19	22	0	17	20	0	89.5	90.9	0.0
2018	14	19	0	13	18	0	92.9	94.7	0.0

2019	15	18	1	13	14	1	86.7	77.8	100.0
2020	20	26	1	19	25	1	95.0	96.2	100.0
2021	13	19	0	12	18	0	92.3	94.7	0.0
2022	10	11	1	6	7	0	60.0	63.6	0.0
2023	9	10	0	7	8	0	77.8	80.0	0.0

Supplementary Table 2. Annual Pufferfish Catch and Import Volumes in Japan (2004-2023).

Year	Pufferfish Catch (tons)	Pufferfish Imports (tons)	total(tons)
2004	6704	12243	18947
2005	6266	10116	16382
2006	5693	11283	16976
2007	5052	7833	12885
2008	5207	6915	12122
2009	4184	5439	9623
2010	4954	6543	11497
2011	6286	4546	10832
2012	5803	5413	11216

2013	4841	4719	9560
2014	4828	4993	9821
2015	4885	5656	10541
2016	4979	4750	9729
2017	4420	4931	9351
2018	4947	3477	8424
2019	4960	3253	8213
2020	3845	2607	6452
2021	6172	2256	8428
2022	6266	2066	8332
2023	4200	2152	6352

Supplementary Table 3. Total Number of Incidents, Patient Cases, and Deaths of Home-based Poisoning Cases from 2004 to 2023 by 5-year Intervals.

Year	Number of Incidents	Number of Patients	Number of Deaths	Number of Home-based Incidents	Number of Home-based Patients	Number of Home-based Deaths	Home-based Incidents Ratio (%)	Home-based Patients Ratio (%)	Home-based Deaths Ratio (%)
2004-2008	178	243	11	131	164	9	73.6%	67.5%	81.8%
2009-2013	98	144	2	74	96	0	75.5%	66.7%	0.0%
2014-2018	102	151	2	84	105	1	82.4%	69.5%	50.0%
2019-2023	67	84	3	57	72	2	85.1%	85.7%	66.7%

Supplementary Figure 1. Geographic distribution of the total number of pufferfish poisoning cases by prefecture in Japan, 2004–2023.

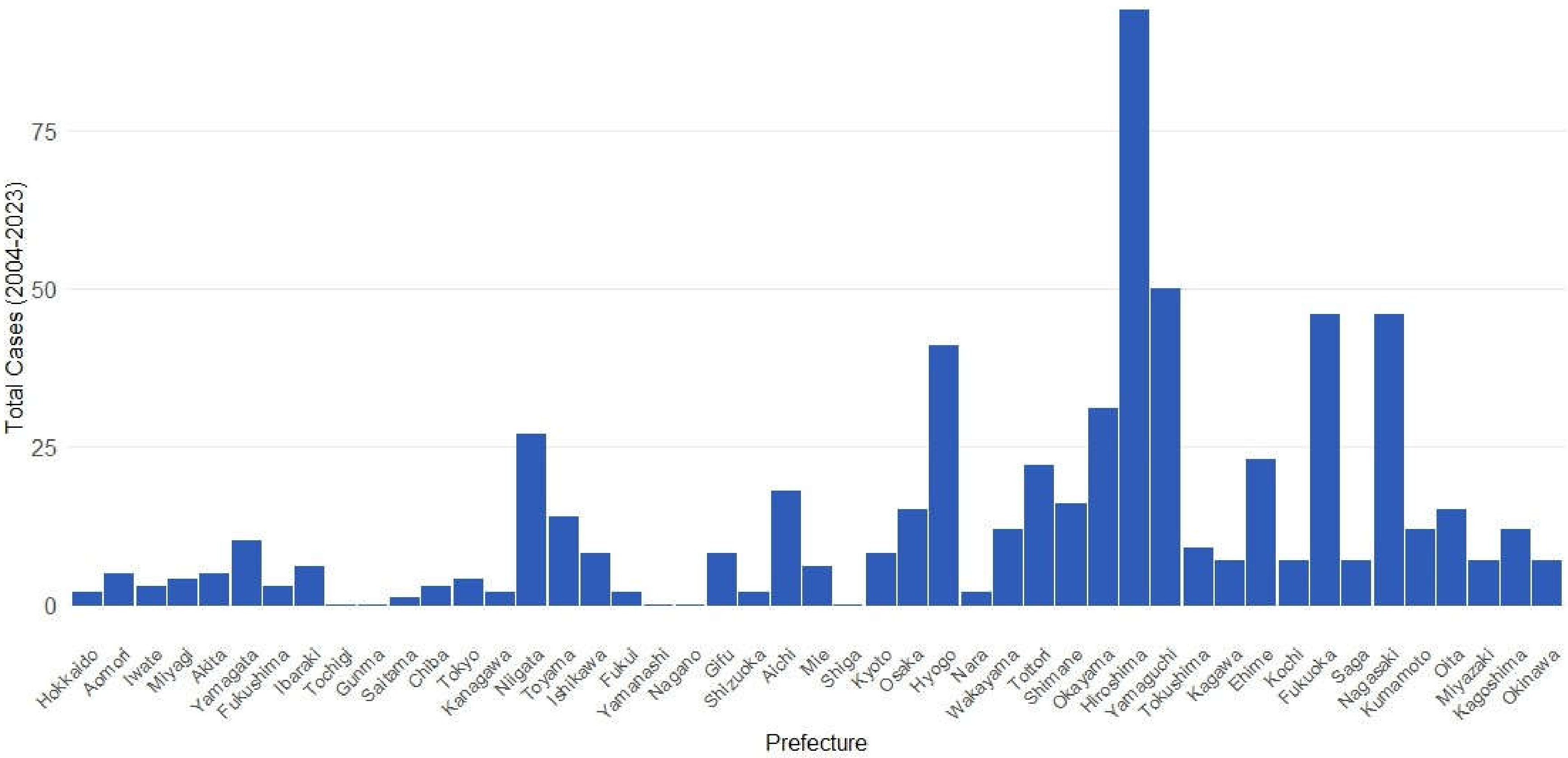

Supplementary Figure 2. Geographic distribution of the average incidence rates of pufferfish poisoning per 1,000,000 population by prefecture in Japan, 2004–2023.

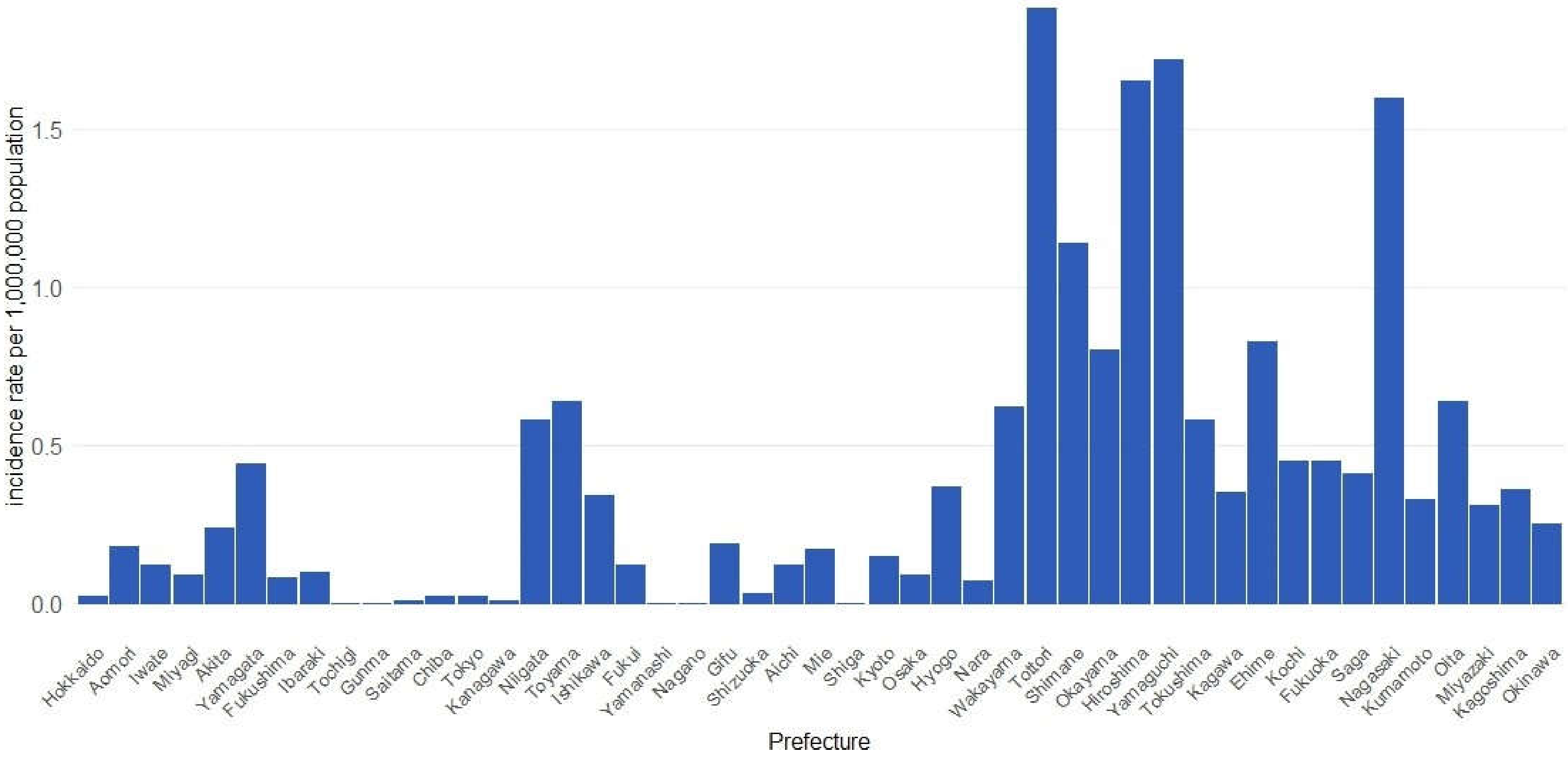

Supplement: Supplementary file 1 [file 2433-3298-9-5-1224-s001.pdf]
